# Modeling the Time Evolution of the Nanoparticle-Protein Corona in a Body Fluid

**DOI:** 10.1371/journal.pone.0010949

**Published:** 2010-06-03

**Authors:** Daniele Dell'Orco, Martin Lundqvist, Cecilia Oslakovic, Tommy Cedervall, Sara Linse

**Affiliations:** 1 Institute of Biology and Environmental Sciences, Section of Biochemistry, University of Oldenburg, Oldenburg, Germany; 2 Department of Biophysical Chemistry, Centre for Molecular Protein Science, Chemical Centre, Lund University, Lund, Sweden; 3 Department of Laboratory Medicine, Section of Clinical Chemistry, Lund University, University Hospital Malmö, Malmö, Sweden; 4 Department of Biochemistry, Centre for Molecular Protein Science, Chemical Centre, Lund University, Lund, Sweden; Instituto de Tecnologia Química e Biológica, Portugal

## Abstract

**Background:**

Nanoparticles in contact with biological fluids interact with proteins and other biomolecules, thus forming a dynamic corona whose composition varies over time due to continuous protein association and dissociation events. Eventually equilibrium is reached, at which point the continued exchange will not affect the composition of the corona.

**Results:**

We developed a simple and effective dynamic model of the nanoparticle protein corona in a body fluid, namely human plasma. The model predicts the time evolution and equilibrium composition of the corona based on affinities, stoichiometries and rate constants. An application to the interaction of human serum albumin, high density lipoprotein (HDL) and fibrinogen with 70 nm N-iso-propylacrylamide/N-tert-butylacrylamide copolymer nanoparticles is presented, including novel experimental data for HDL.

**Conclusions:**

The simple model presented here can easily be modified to mimic the interaction of the nanoparticle protein corona with a novel biological fluid or compartment once new data will be available, thus opening novel applications in nanotoxicity and nanomedicine.

## Introduction

When nanoparticles enter a biological fluid, proteins and other biomolecules compete for the nanoparticle surface, thus leading to the formation of a protein corona that defines the biological identity of the particle [1]. The corona is dynamic in nature and its composition varies over time due to continuous protein association and dissociation events. Eventually equilibrium is established, at which point the concentrations of free and bound proteins are such that for each protein the association and dissociation rates have become equal and the continued exchange will not affect the composition of the corona. However, the corona will again change composition if the nanoparticle moves to another compartment or fluid [2]. Particle material, size and surface properties have been found to play a significant role in determining the composition of the corona [1]–[4]. The term “hard corona” defines the long-lived equilibrium state, representing a fingerprint of a nanoparticle in a certain environment [2]. Understanding the time evolution of the protein corona in a given biological fluid, and in particular the time-scales on which particle-associated proteins exchange with free proteins, enables predictions of the particle's fate as regards interactions with specific receptors or predictions of its life time in blood. Such understanding will have impact both on nanomedicine and on nanotoxicity.

In this work, we present a simple and effective mathematical model that describes the kinetics of the corona formation around a copolymer nanoparticle based on experimentally determined association and dissociation rate constants. This is to our knowledge the first attempt to model the competitive nanoparticle-protein interaction kinetics that contributes to the formation of the corona. Parameters determined in our previous studies [1]–[6] and by surface plasmon resonance (SPR) measurements performed in this study, as well as data taken from the literature, were introduced in the model to describe the interaction of three human plasma proteins with copolymer nanoparticles. The nanoparticles used are synthetic N-iso-propylacrylamide/N-tert-butylacrylamide (NIPAM/BAM) 50∶50 copolymer nanoparticles of 70 nm diameter. The three proteins investigated are human serum albumin (HSA), high density lipoprotein (HDL) and fibrinogen (Fib). These proteins were chosen because they have been found to be major biological macromolecules in the corona around the copolymer particles [1]–[4]. HDL is a complex composed of different proteins, phospholipids, cholesterol and triglycerides. Cedervall et al. showed that the corona around the copolymer particles contains the apolipoproteins that build up HDL [1], [3], and Hellstrand et al. identified phospholipids in the corona in a range that suggests that the intact HDL particle is interacting with the copolymer nanoparticles [4]. The three proteins are found in human blood at different concentrations, see **Table 1**, and have different molecular sizes.

**Table 1:** Chemical, geometric and kinetic/thermodynamic parameters used in the model.

	Concentration in blood (mg/mL)[a]	Radius (nm)[b]	Initial Concentration ([M])[c]	*k^on^* (M^−1^ s^−1^)[d]	*k^off^* (s^−1^)[e]	K_A_ (M^−1^)[f]	n_i_ [g]
***NP***	-	35	18×10^−9^	-	-	-	-
***HDL***	3[h]	5	1.5×10^−5^	3×10^4^	3×10^−5^	1×10^9^	256
***HSA***	40[i]	4	6.0×10^−4^	2.4×10^3^	2.0×10^−3^	1.2×10^6^	380
***Fib***	3[i]	8.3	8.8×10^−6^	2.0×10^3^	2.0×10^−3^	1.0×10^6^	109

[a] average concentration found in human blood.

[b] protein radius calculated as if each protein were spherical.

[c] starting concentration in the simulation.

[d] association rate constant; see Materials and Methods section for details.

[e] dissociation rate constant: see Materials and Methods section for details.

[f] equilibrium constant (K_A_ = *k^on^*/*k^off^*).

[g] ideal number of binding sites on the nanoparticle surface for the protein *i*.

[h] see Materials and Methods section for details.

[i] average values for a healthy adult human [18]–[20].

## Results and Discussion

The number of binding sites on a nanoparticle will be determined by the size of the particle as well as of the protein. However, the hydrophobicity of the nanoparticle strongly influences its protein binding properties [1], [5]. Differences in the nanoparticle surface chemistry may control the number of possible binding sites. It is thus important to include in the model a factor that accounts for the maximum number of available binding sites on the nanoparticle surface for each specific protein. This is expected to affect both the competition between different proteins for the same nanoparticle and the maximum level of coverage of the nanoparticle surface by each specific protein. Considering as a first approximation each bound protein as a sphere physically in contact with the nanoparticle surface, the ideal number of binding sites *n_i_* for the protein *i* is given by the ratio between the extended nanoparticle surface and the cross-section of a protein:
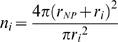
(1)where *r_NP_* is the nanoparticle radius, here 35 nm, and *r_i_* is the protein radius if proteins are considered as spheres. The extended rather than actual nanoparticle surface better describes the available surface, since CD data indicate that proteins adsorb to the copolymer nanoparticles in a native-like fashion (unpublished data), so that the area of interest is the one that is one protein radius above the nanoparticle surface.

The number of binding sites per protein type was included in the association rate laws of each protein-nanoparticle reaction, to serve as correction factors accounting for the maximum surface occupancy of each protein. The geometrical quantities are reported in **Table 1**, which further lists all the parameters employed in the simulations.

In our previous experiments on HSA binding to the same type of nanoparticles we found that the binding data were well fitted by a simple binding model devoid of any apparent cooperativity or inter-protein interaction phenomena [1]. Assuming that the same mechanisms remain valid also for the other two proteins, the reversible biochemical equations describing the dynamics of the nanoparticle (NP)-protein interactions can be modeled as follows:
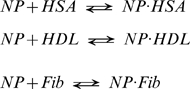
(2)


With the assumption of law of mass action kinetics, the biochemical system can be modeled by a set of ordinary differential equations (ODEs), and the time evolution of each protein-nanoparticle complex can be assessed by numerically solving the following ODE system:
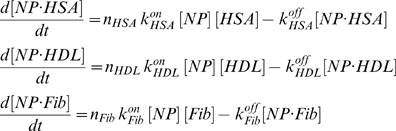
(3)


The details of the model implementation and simulations are given in the Material and Methods section.

**Figure 1:**
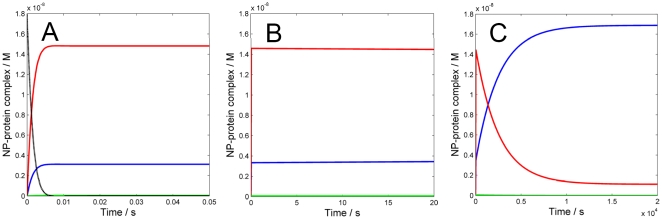
Numerical simulation of the time evolution of the interaction between three human plasma proteins and 50∶50 NIPAM/BAM NP with 70 nm diameter. The values used for rate constants and correction factors accounting for the maximum number of binding sites on the NP surface per protein are given in Table 1. The concentrations of NP (black-dotted), NP⋅HSA (red), NP⋅HDL (blue) and NP⋅Fib (green) as a function of time are shown. A) First 0.05 s and B) first 20 s of simulation: the concentrations of free NP and proteins decrease fast (t<10 ms) due to the rapid formation of NP⋅protein complexes. Initially HSA is the major contributor to the corona composition (80.7%) followed by HDL (18.9%) and Fib (0.4%). C) First 20,000 s of simulation: a stable equilibrium is reached in about 4 hours, in which HDL is the most abundant protein in the corona (93.6%) followed by HSA (6.4%).

The results of our simulations are shown in **Figure 1**. When the evolution of the system is monitored for a relatively short time, two distinct temporal regimes are observed. During the very fast initial transient events (t<<0.05 s, Figure 1A), the concentration of the free nanoparticles (NP) dramatically decreases due to the fast formation of complexes between NP and each of the three proteins. The concentration of these complexes appears fairly stable in the first 20 s (Figure 1B). At t = 10 s, 80.7% of the available NP surface is covered by HSA, 18.9% by HDL and only 0.4% by Fib (Figure 1B). However, if the simulation is run for a longer time, a very different scenario takes over (Figure 1C). During the following ∼14,000 s, corresponding to 3.9 h, a redistribution of proteins at the NP surface is taking place, i.e. the corona composition changes. After 3.9h, equilibrium is reached and according to the simulation, 93.6% of the protein corona is constituted of HDL, and the remaining 6.4% is HSA. Fib does not significantly contribute to the corona, even at longer equilibration times.

**Figure 2:**
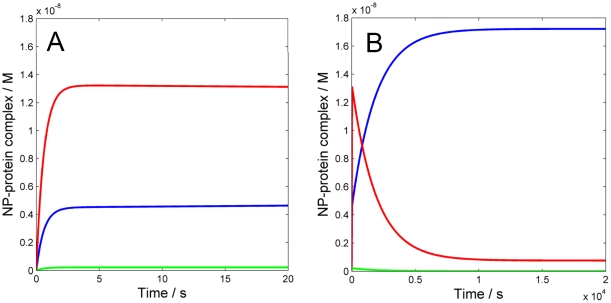
Numerical simulation of the time evolution of the interaction between three human plasma proteins and 50∶50 NIPAM/BAM NP with 70 nm diameter without the correction factors accounting for the maximum number of binding site on the NP surface per protein (all *n_i_*'s = 1). The rate constants used are given in Table 1. The concentrations of NP⋅HSA (red), NP⋅HDL (blue) and NP⋅Fib (green) as a function of time are shown. A) First 20 s of simulation: the initial transitory phase develops slower than in the case shown in Figure 1. B) First 20,000 s of simulation: the final equilibrium is reached faster compared to the case shown in Figure 1 and with a slightly different final composition of the corona.

**Figure 3:**
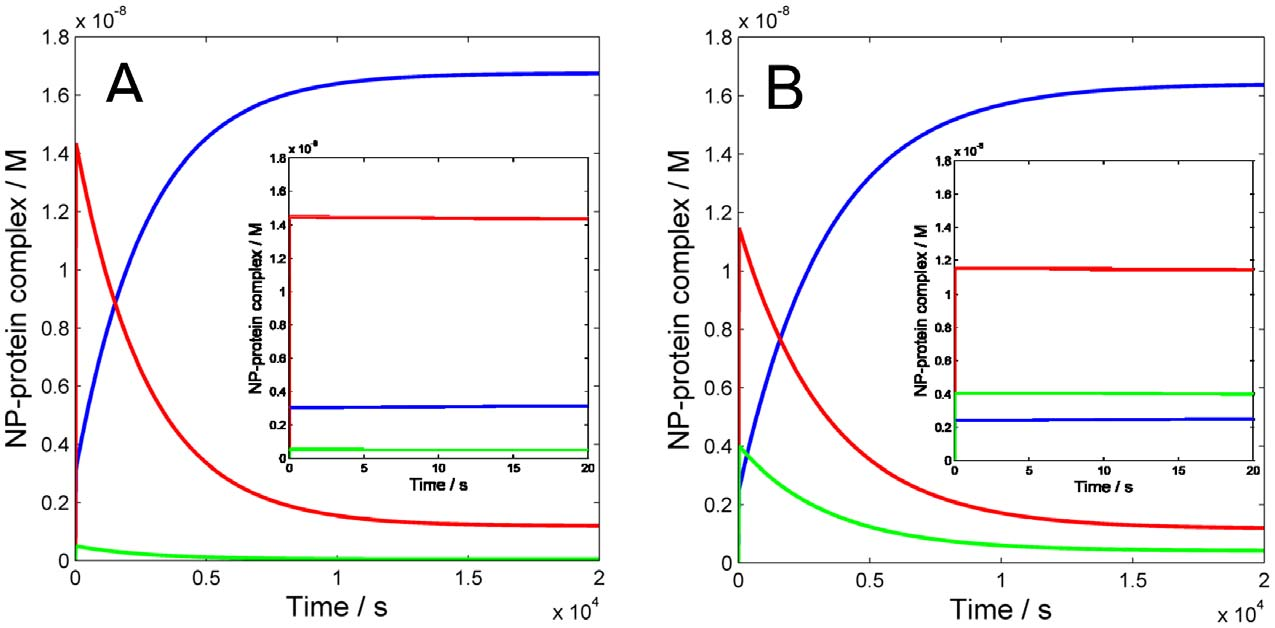
Numerical simulation of the time evolution of the interaction between three human plasma proteins and 50∶50 NIPAM/BAM NP with 70 nm diameter with different number of binding sites for Fib. The values used for rate constants and correction factors accounting for the maximum number of binding sites on the NP surface for HSA and HDL are given in Table 1. The number of maximum binding sites for Fib on the NP surface are A) n_Fib_ = 1090 (10-fold increase) and B) n_Fib_ = 10,900 (100-fold increase). The concentrations of NP⋅HSA (red), NP⋅HDL (blue) and NP⋅Fib (green) as a function of time are shown. The inset in each panel reports the first 20 s to show the predicted composition of the early corona.

The correction factors *n_i_*'s exert an effect on the overall kinetics in the early transitory phase. This effect is removed if all such factors are set equal to one, which corresponds to the case in which all proteins are assumed to have the same number of binding sites (or maximum coverage) on the NP surface. **Figure 2A** shows that the initial transitory phase is then slowed compared to the case of distinct *n_i_*'s, and it would take about 3 s to reach the first pseudo-stable state. The same pattern as in Figure 1B is observed for the early corona composition, except that both HDL (25.4%) and Fib (1.2%) reach slightly higher levels. Interestingly, running the simulations for a longer time shows that the final equilibrium is reached faster, in ∼10,800 s (3 hours), and the final levels (95.5% of HDL and 4.4% of HSA, while Fib again is predicted to exert a negligible effect) are similar to those found with different *n_i_*'s. Recently, we experimentally investigated the role of NP size on the NP-protein interaction, using the same NIPAM/BAM nanoparticles (70 to 700 nm in diameter) and plasma. While the amount of bound protein varied with particle size and scaled with the available surface area, the protein stoichiometry per NP surface area was found to be the same for all sizes, except for a small reduction observed for the smallest particles with 70 nm diameter [3], [5], indicating that surface curvature is not a major determinant for the relative affinities of biological entities such as proteins and apolipoproteins for the NIPAM/BAM copolymer particles over the investigated size range. The results of the simulations presented here, especially the limited dependence of *n_i_*'s for the observed protein pattern at the final equilibrium, suggest that the relative size of protein with respect to the particle mainly influences the time required to reach the equilibrium. We should also point out that the spherical approximation might be too rough, especially for fibrinogen, which is an elongated protein. Recent data concerning other nanoparticles [7], [8] seem to support the hypothesis that Fib binds to the nanoparticle surface with its short ends, hence reducing the effective cross-section in favor of increased maximum number of binding sites. To probe the effect of this different binding mode on the overall corona dynamics we performed two sets of simulations with n_Fib_ increased 10- and 100-fold, respectively, relative to the previous value (**Figure 3**). When the number of binding sites for Fib is increased 10-times (Figure 3A), no significant difference is observed on the short and long-time course with respect to the original case shown in Figure 1. The main difference is in fact a slightly increased level of Fib concentration in the early corona, which however does not perturb the timing and the composition of the hard corona. However, when the number of binding sites for Fib is increased 100-times (Figure 3B), the composition of the early corona is predicted to change significantly. In detail, HSA would cover 63.8% of the NP, while Fib and HDL would cover 22.3% and 13.9% respectively. Hence, in this case, the concentration of nanoparticle-bound Fib is expected to exceed that of NP-HDL in the early corona. The simulations nevertheless suggest that the timing and the composition of the hard corona are only marginally affected, and the process of reaching the equilibrium appears to be slightly slowed down with a final detectable level for Fib (2.3%).

**Figure 4:**
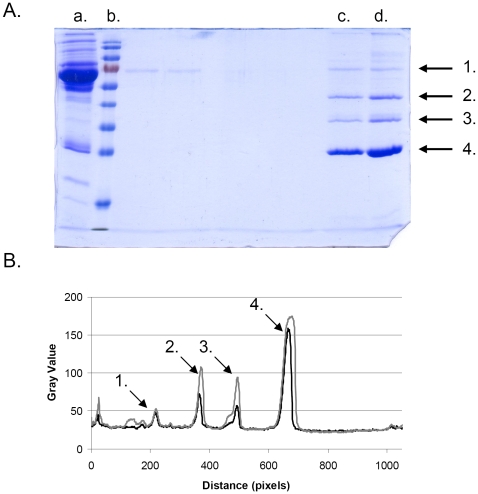
Hard corona around the copolymer particles after 30 s and 6 h of incubation in human plasma. A) 12% SDS-gel showing; a: human plasma diluted 20 times (5 µl added to the gel), b: MW standard, c: copolymer particles incubated 30 s in plasma, d: copolymer particles incubated 6 h in plasma. Arrow 1–4 indicates the gel bands for serum albumin, apolipoprotein A-IV, apolipoprotein E, and apolipoprotein A-I, respectively. B) traces for lane c and d in panel A as obtained using ImageJ (see the Materials and Methods section). The arrows indicate the same proteins as in panel A.

To experimentally characterize the composition of the hard corona formed around a nanoparticle the unbound or weakly bound proteins must first be removed. This is done using a purification scheme that includes several centrifugation and washing steps [3], [5]. The whole process takes ∼15–20 min (for details see Material and Methods). Thus the very early events on a sub-second to minute time-scale can not be experimentally investigated by this technique. Unbound and weakly associated proteins are washed away from the protein-nanoparticle complexes during this process, however, also a fraction of the proteins that participate in building up the hard corona, apolipoproteins in the case of copolymer particles, are washed away in the process. This means that for the very early events it is not possible to make direct correlation between the simulated results and quantitative results obtained experimentally by SDS-PAGE gels. The model shows that the initial corona is mainly built up by HSA, which is not surprising considering the abundance of serum albumin in blood. However, the interaction between HSA and the copolymer particles is relatively weak (Table 1). Pull down experiments, which measure the concentration in the supernatant after the particles have been pelleted, with particles mixed with single protein solutions of HDL, HSA, and apolipoprotein A-I show that both HDL and apolipoprotein A-I concentrations are reduced (i.e. they interact strongly with the particles and are pelleted together with the particles) while the HSA concentration in the supernatant remains unchanged (unpublished data). The interaction between HSA and the copolymer particles is not strong enough for the early, very dynamic, corona to withstand the purification scheme. The corona evolves with time, as the model shows, going from a soft corona dominated by HSA to a hard corona dominated by apolipoproteins (HDL). **Figure 4** shows the hard corona around the copolymer particles after 30 s (+3 min centrifugation) respectively 6 hours incubation in plasma. The amounts of detected apolipoprotein A-I, A-IV and E have increased compared to the amount of HSA for 6 h sample compared to the 30 s sample, in line with the current modeling.

**Figure 5:**
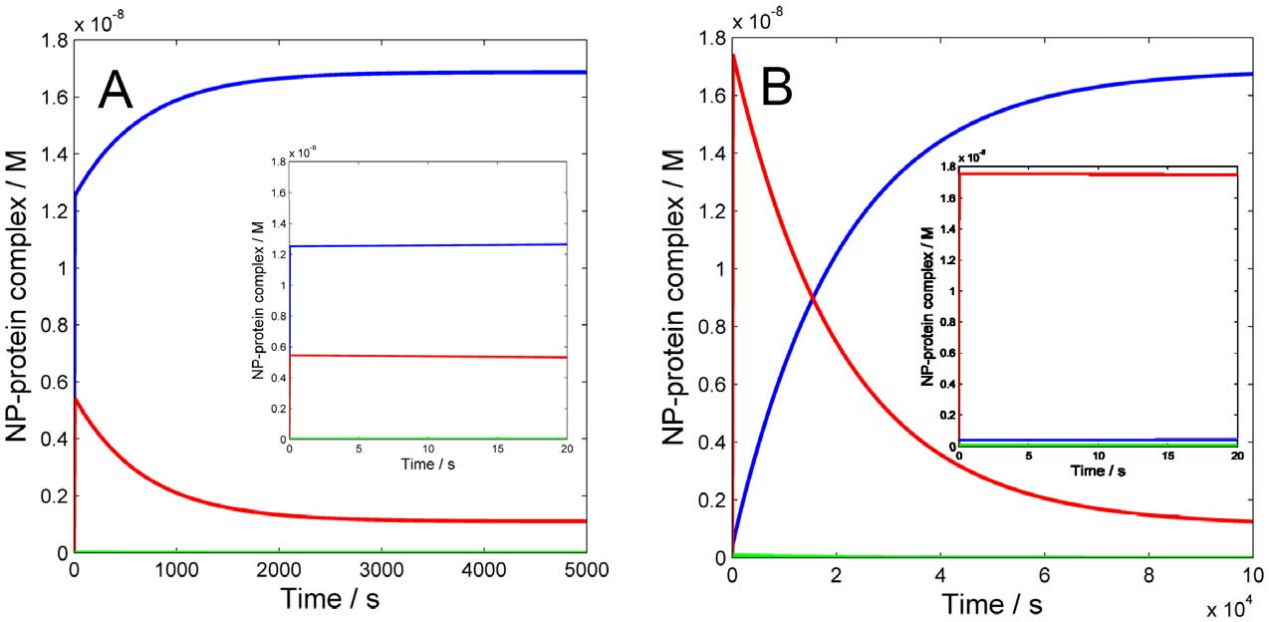
Two simulations of NP-protein interaction kinetics showing the time evolution of the occupancy of HDL (blue), HSA (red) and Fib (green) on the nanoparticle surface, using the same affinity but different kinetics for HDL. The affinity for HDL-NP interaction is 1×10^9^ M^−1^ while that for HSA-NP and Fib are respectively 1.2×10^6^ M^−1^ and 1×10^6^ M^−1^ (Table 1). The association and dissociation rate constants of HDL are modified with respect to the original values (k^on^ = 3×10^4^ M^−1^ s^−1^and *k^off^* = 3×10^−5^ s^−1^) to be 10 times higher in panel A) and 10 times lower in panel B). The insets in each panel show the composition of the early corona (t = 20 s).

The results from the simulations clearly show that the temporal development of the corona strongly depends on the association/dissociation rates of each protein, which in turn determine the time required to reach the equilibrium state. Hence, it is fundamental that the rate constants used in the model arise from directly measured kinetic parameters rather than rely on equilibrium data such as affinity measurements. For the simulations in this work, the association and dissociation rate constants were experimentally determined for HDL, HSA and Fib (see the Materials and Methods section). **Figure 5** illustrates the importance of distinguishing between kinetic and equilibrium data and shows how a change in *k^on^* and *k^off^* values, at constant K_A_, affects the time to reach equilibrium. By using the experimentally determined *k^on^* and *k^off^* values the affinity for HDL is calculated to be 10^9^ M^−1^ (Table 1). However, different combinations of kinetic constants yield the same affinity. Figure 5A shows the simulated case with a 10^9^ M^−1^ HDL-NP affinity, but in this case both *k^on^* and *k^off^* are assumed to be 10-fold higher compared to the experimentally determined values (Table 1). The graph inset shows a completely different early corona, in which HDL already prevails over the two other protein types. Moreover, while a very similar corona composition is expected at equilibrium (compare with equilibrium levels in Figure 1B), the modified kinetics allow the equilibrium to be reached much faster, in about 3000 s (50 min). On the other hand, using the same affinity but lowering both *k^on^* and *k^off^* 10 times, leads to a completely different scenario (Figure 5B). The early corona would be almost entirely formed by HSA (see inset) and the same final equilibrium, similar to the one predicted with experimentally determined parameters (se Figure 1B), would be reached much slower, in ∼10^5^ s (28 hours). Hence, in order for the simulations to be realistic we conclude that it is essential to collect robust experimental data that are not limited to affinity measurements but include direct assessment of kinetic parameters.

Overall, the results of the simulations presented in this work are consistent with our recent experimental findings. Indeed, by SDS-page we showed that HSA, despite its higher concentration that is reflected by initially more abundant binding, is soon replaced by the higher-affinity and slower-exchanging apolipoproteins AI, AII, AIV and E [3] (which all come from the interaction of HDL with the copolymer particles) [5]. We further observed that the interaction with HDL is specific, as HDL binds to copolymer nanoparticles with much higher affinity than other lipoproteins, like low density lipoprotein or very low density lipoprotein, probably mediated by apolipoprotein A-I, which is its major component [4]. Hence, the results of our simulations (Figure 1) rationalize and quantitatively confirm these prior observations.

The advantage of the simple model presented here is that it can easily be modified to mimic, for instance, the interaction of the NP corona with a novel biological fluid or compartment once new data will be available. In that case the novel initial conditions would correspond to the equilibrium state outlined from the simulations presented here. By adding novel molecular species to the model, the time evolution of the whole system can be monitored and the results of simulations experimentally tested. Moreover, once a proper overall kinetic description is achieved for the corona around a NP in a certain biological environment, the whole model could be inserted into a biochemical pathway and used to predict the perturbations exerted by the corona on that pathway. Very recently the procedure used to build the model presented here has been successfully tested in a novel bottom-up strategy for simulating a complex signal transduction pathway such as that of phototransduction in vertebrate cells [9] indicating that when sufficient experimental information is available it is possible to bridge between the biochemical and cellular levels, thus increasing the relevance and the potential of modeling for general nanomedicine and systems biology purposes.

Nanoscience is expected to deeply affect medicine and biotechnology in the near future. Solid modeling procedures based on validated techniques are expected to provide valuable insights into the molecular mechanisms of nanoparticle-cell/tissue interactions and sound predictions of novel properties and applications.

## Materials and Methods

### Surface Plasmon Resonance spectroscopy: measurement of the kinetic parameters for the NP-HDL interaction

Surface plasmon resonance was employed to study HDL association to and dissociation from the nanoparticle. HDL was used in the form of reconstituted HDL (rHDL) particles. rHDL was prepared as previously described [10]. Briefly, phospholipids (phosphatidylcholine) were dried and resuspended in PBS buffer with *n*-octyl-*β*-D-glucopyranoside. Solubilized lipids were mixed with apoA–I (30∶1 phospholipid/apoA–I molar ratio) and dialyzed.

A BIAcore 3000 instrument (General Healthcare Life Science TM) was used and the experimental conditions were the same as in our previous work [1] in order to allow a direct comparison between the newly measured kinetic parameters and those of other protein-nanoparticle interactions previously characterized. In brief, thiol-linked nanoparticles were dissolved in 20 mM sodium phosphate buffer,100 mM NaCl (pH 7.5), on ice and applied to gold surface. Alkylated (to avoid coupling to gold via free thiols groups) single proteins diluted in flow buffer (10 mM Tris/HCl, pH 7.4, 3 mM EDTA, 150 mM NaCl, 0.005% Tween-20) were injected for 30 min to study the association kinetics. After 30 min, buffer was flowed over the sensor chip surface for 10–24 h to study dissociation kinetics. Results from kinetics experiments are reported in **Table 1**.

### Human plasma

Human blood was withdrawn from seemingly healthy humans, who all signed a consent stating that their blood could be used in scientific research projects, into vessels pre-treated with EDTA-solution. The blood vessels where centrifuged for 5 min at 800 RCF. The supernatants (the plasma) were transferred to new vessels and stored in −80°C freezer until time of use. Before use, the plasma vessel was thawed and centrifuged for 3 min at 16.1 kRCF and the supernatant was transferred to a new vessel. Lund University Hospital ethic committee has approved all experiments in this article conducted with human plasma.

### Detection of the hard protein corona

Identification of protein in the hard corona was performed as previously reported [3] and described below. Lyophilized 70 nm copolymer (NIPAM∶BAM 50∶50) particles were dissolved on ice in PBS (10 mM phosphate, 150 mM NaCl, 1 mM EDTA, pH 7.5) to a concentration of 10 mg/mL. The particle solution was kept on ice throughout the experiment. 800 µL of human plasma was mixed with 100 µL of the particle solution. The mixture was incubated for 30 s (first sample) or 6 h (second sample) followed by centrifugation at 15 kRCF for 3 minutes. The plasma was discarded, the pellet resuspended in 1 mL PBS, and the solution was transferred to a new vessel and centrifuged as above. This washing step was repeated three times with change of vessel each time to avoid artifacts from protein bound to the vessel wall. Proteins were desorbed from the nanoparticles in the final pellet by adding 30 µL SDS sample buffer and incubating at 100°C for 5 minutes, after which 10 µL were applied to SDS PAGE (**Figure 4**). The intensity of each protein band was estimated through digital gel scan using ImageJ software [11] (http://rsb.info.nih.gov/ij/), and proteins identified by mass spectrometry [1] (**see** **Table 2**). The experiments were repeated six times with comparable results, and Figure 4 reports a representative outcome from such experiments.

**Table 2:** Integrals[a] for the peaks in Figure 4B.

Peak	Intensity (30 s sample)[b]	Intensity (6 h sample)[b]	Protein[c]
1	653	652	Serum Albumin
2	1487	3137	Apolipoprotein A-IV
3	1157	3019	Apolipoprotein E
4	8664	15979	Apolipoprotein A-I

[a] Integrals calculated with ImageJ (http://rsb.info.nih.gov/ij/, see Materials and Methods).

[b] The time refers to incubation time for copo1lymer particles in plasma.

[c] Protein identification from Cedervall et al, Ref. [3].

### Model building and simulations

A kinetic model of the nanoparticle-protein corona was built by combining the rate equations for association and dissociation of proteins with nanoparticle, with concentrations, individual values of *k^on^*, *k^off^* and maximum stoichiometry at full occupancy in a single layer as described in Table 1. The building of the kinetic model was carried out by means of SBTOOLBOX2 for Matlab (http://www.sbtoolbox2.org) [12]. This tool allows specifying the model directly through biochemical reaction equations that can then be converted into the ODEs to be numerically solved. All numerical simulations in this work were carried out within the framework of the SBTOOLBOX2 following an automatic generation of compiled C-code models, based on the CVODE integrator from SUNDIALS [13].

### Calculation of the HDL concentration in blood

High density lipoprotein (HDL) is defined as the lipoprotein fraction with a density range between 1.063 to 1.21 g/mL. The HDL fraction comprises two major fractions: HDL2 (1.063–1.125 g/mL) and HDL3 (1.125–1.21 g/mL). HDL2 and HDL3 have different sizes and molecular weights [14]. They are composed of complexes of proteins, cholesterol, phospholipids, cholesteryl ester, and triglycerides [15]. The HDL concentration is normally indirectly measured via the concentration of cholesterol, either for total HDL or for each sub fraction. In this work the HDL concentration was used in the simulation, since Hellstrand et al. [4] have shown that HDL binds to the copolymer particles. The HDL concentration was calculated by using the cholesterol molecular weight, cholesterol content values [15] for HDL2 and HDL3, and reported levels of HDL2- and HDL3-cholesterol for healthy control groups [11], [16]–[17].
